# Synthesis and Effect on Human HepG2 Cells of 1,2-bis- (2-Methylallyl)disulfane

**DOI:** 10.3390/molecules15053634

**Published:** 2010-05-18

**Authors:** Chunxiao Ji, Fenglian Ren, Jun Dai, Ming Xu

**Affiliations:** 1 College of Chemistry and Chemical Engineering, Central South University, Changsha, Hunan 410083, China; E-Mail: renfl2009@yahoo.com.cn (F.R.); 2 Research Institute for Molecular Pharmacology and Therapeutics, Central South University, Changsha, Hunan 410083, China

**Keywords:** 1,2-bis(2-methylallyl)disulfane, identification, apoptosis

## Abstract

1,2-bis(2-methylallyl)disulfane was synthesized from sodium sulfide and 3-chloro-2-methylpropylene. The structure of the target product was confirmed by GC-MS, ^1^H-NMR and elemental analysis. Cell viability assay, flow-cytometric analysis and protein expression results showed that 1,2-bis(2-methylallyl)disulfane could significantly inhibit the proliferation, and induce the apoptosis of human HepG2 cells.

## 1. Introduction

Garlic is a kind of liliaceae allium, and allicin is the general name for the main bioactive components of garlic, which is a mixture of a variety of allyl sulfides, including diallytrisulfide compounds (DATS), diallyl disulfide compounds (DADS) and many kinds of thioethers. Allicin has a variety of biological activities [1,2,3,4,5,6]. At present, there is an abundance of research on various aspects of allicin, and those studies have shown that the DADS, DATS and diallyl sulfur compounds (DAS) in allicin have anti-tumor effects and can inhibit the growth of various tumor cells, such as human colon cancer cells, human liver cells, human gastric cancer cells, and so on [7,8,9,10,11]. 1,2-bis(2-Methyl- allyl)disulfane exists in natural garlic, and it is one of the bioactive components of allicin, but its content is not very high [12]. In this paper, we have successfully synthesized 1,2-bis(2-methylallyl)- disulfane using sodium sulfide and 3-chloro-2-methylpropylene as raw materials, and proved that 1,2-bis(2-methylallyl)disulfane has an antitumor effect.

## 2. Results and Discussion

### 2.1. Structural characterization of 1,2-bis(2-methylallyl)disulfane

#### 2.1.1. GC-MS analysis results

The compound was analyzed using GC-MS and the results are quite consistent with the structure of 1,2-bis(2-methylallyl)disulfane. The data are shown in Table 1. 

**Table 1:** GC-MS analysis results.

No.	Peaks detected in GC-MS ( *m/z*)	Possible groups	Accurate *m/z* value for the proposed groups
1	39-43	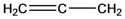	40
2	41-43	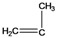	41
3	55	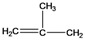	55
4	57	Not characterized	
5	71	Not characterized	
6	81	Not characterized	
7	86-88	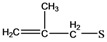	87
8	113	Not characterized	
9	118-121	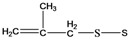	119
10	133	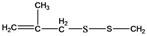	133
11	174	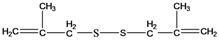	174

#### 2.1.2. ^1^H-NMR analysis of 1,2-bis(2-methylallyl)disulfane

**Figure 1:**
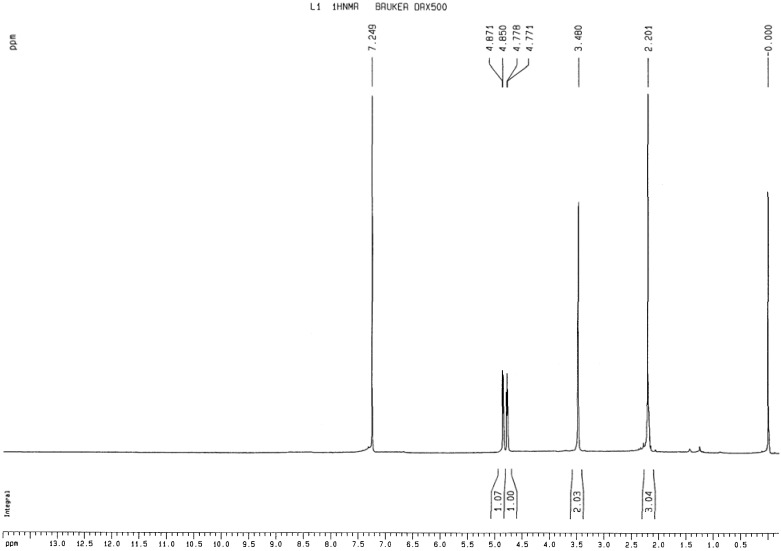
^1^H-NMR spectrum of 1,2-bis(2-methylallyl)disulfane.

The compound was analyzed by NMR under the optimum conditions using TMS as the standard and using the value of 7.249 ppm for the water peak, as shown in Figure 1. The =CH_2 _group appeared as a split peak because the two hydrogen atoms are in a *cis-trans* relationship (Figure 1). The results are in agreement with the structure of 1,2-bis(2-methylallyl)disulfane.

#### 2.1.3. Elemental analysis of 1,2-bis(2-methylallyl)disulfane

The structure was also confirmed by elemental analysis, and the results are shown in Table 2. The results were in good agreement with the formula of 1,2-bis(2-methylallyl)disulfane(C_8_H_14_S_2_).

**Table 2:** The elemental analysis results of 1,2-bis(2-methylallyl)disulfane.

Elemental	C	H	S
Found (%)	54.93	8.01	36.01
Calculated (%)	55.12	8.09	36.79

### 2.2. The effect of 1,2-bis(2-methylallyl)disulfane on human HepG2 cells

#### 2.2.1. Cell activity

**Figure 2:**
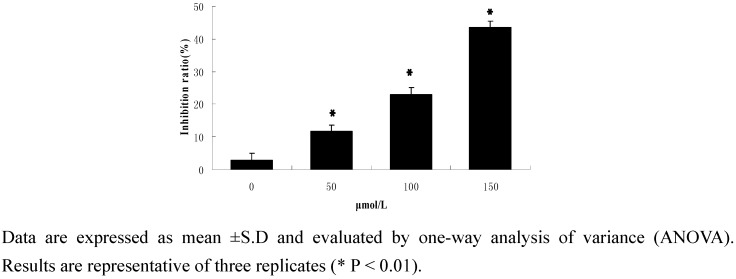
MTT assay results.

Cell viability was determined by the MTT assay. As shown in Figure 2, when human HepG2 cells were exposed to drug concentrations of 0, 50, 100 and 150 μmol/L for 24 h, the corresponding dose-dependent inhibition ratios were 11.63%, 23.01% and 43.47%, respectively. (Figure 2).

#### 2.2.2. Flow-cytometric analysis of apoptosis

**Figure 3:**
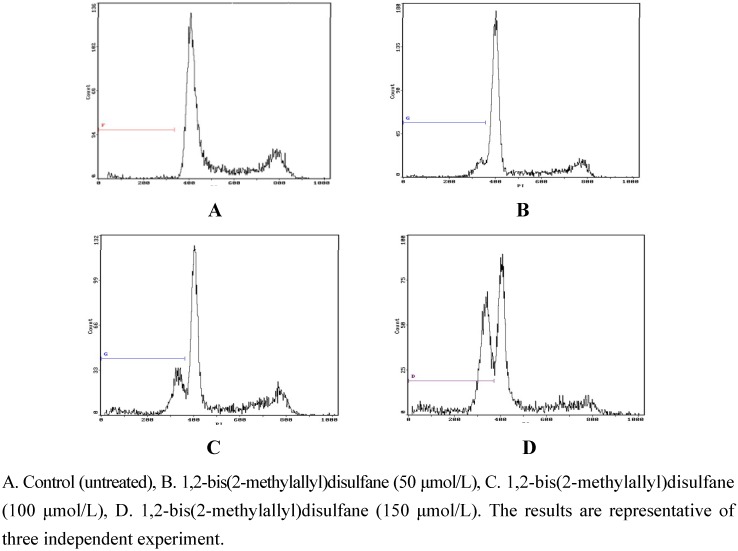
Effects of each group on apoptosis in in human HepG2 cells.

**Figure 4:**
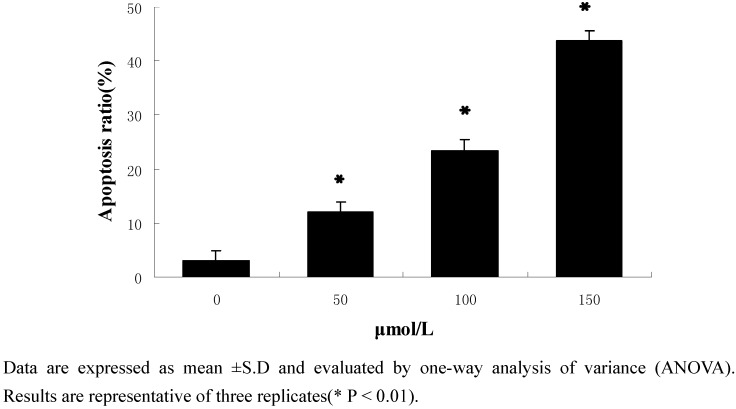
Results of the flow cytometry analysis.

To further examine the effects of 1,2-bis(2-methylallyl)disulfane on apoptosis, flow cytometry was used to quantify the apoptotic state (Figure 3 and Figure 4). After treatment and incubation for 24 h, the apoptosis ratio of cells treated with 50 μmol/L, 100 μmol/L and 150 μmol/L 1,2-bis(2-methylallyl)disulfane was 11.97%, 23.49% and 43.69% respectively. The results also supported the notion that 1,2-bis(2-methylallyl)disulfane induced apoptosis of HepG2 cells in a concentration-dependent manner.

#### 2.2.3. Protein expression

**Figure 5:**
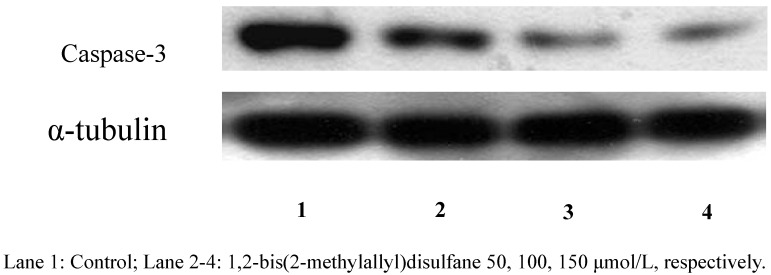
Effect of 1,2-bis(2-methylallyl)disuelfane on the protein expression by Western blot.

**Figure 6:**
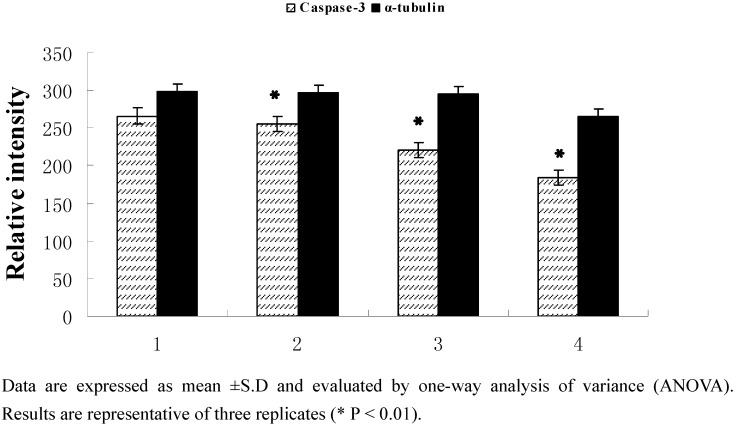
Protein expression results.

After treatment with 1,2-bis(2-methylallyl)disulfane (50, 100, 150 μmol/L, respectively) for 24 h, the caspase-3 zymogen protein bands became thinner (Figure 5). Studies have proved that the unactivated caspase-3 will trigger apoptosis when it is activated and play a very important role as the central effector of apoptosis when cells start apoptosis. Our results showed that 1,2-bis(2-methylallyl)- disulfane could significantly enhance the activity of caspase-3 (Figure 6). 

## 3. Experimental

### 3.1. General

3-Chloro-2-methylpropylene, 1-bromo-4-methyl-2-amylene and 1,2-dimethoxyethane (DME) were purchased from Fluka; ether, benzophenone, sulphur, chloroform and carbinol were purchaed from Nanjing Chemical Reagents Co. MTT (3-(4,5dimethylthiazol-2-yl)-2,5-diphenyltetrazolium bromide) and propidium iodide (PI) were purchased from Sigma. Caspase-3 and α-tubulin were purchased from Cell Signaling. DME was dried with metal sodium using benzophenone as the indicator before being used. ^1^H-NMR was recorded on a Bruker DRX-500 spectrometer at 298K, elementary analysis was performed on a Perkin-Elmer 240 C analytic instrument. GC-MS analyses were performed on a HP6890 gas chromatograph equipped with a HP 5973 mass selective detector (MS), equipped with a fused-silica-capillary column, DB-5, (30 m × 0.32 mm).

### 3.2. Synthesis of 1,2-bis(2-methylallyl)disulfane

The synthetic route is shown in Scheme 1. Metallic sodium sheet (12.7 g, 0.55 mol) was added to DME (100 mL) with fast stirring, and then sulphur powder (16.0 g, 0.50 mol) added after the sodium metal was completely dispersed, and the mixture was stirred at the room temperature for 2 h, until the Na_2_S_2 _was formed. The product was kept sealed in cool place [13,14,15]. Na_2_S_2_(50 mL) was added into a round bottom flask (250 mL), then DME (50 mL) which contained 3-chloro-2-methylpropylene (45 g, 0.50 mol) was added dropwise to the round bottom flask with continuous stirring, and the mixture was reacted in a 70 ºC oil bath for 4 h. After completing the reaction, the solvent was removed by rotary evaporator to give a bright yellow oily substance. The oily product was added into distilled water (50 mL) and dispersed with ultrasound, then extracted with ether (20 mL × 5), and extracted with chloroform three times. The combined extracts were evaporated under vacuum to remove the solvent. Then using chloroform/methanol (v/v = 95/5) as mobile phase, the product was purfied by silica gel column chromatography[16,17,18,19,20]. A yllow oil was obtained (56.86 g, 65.3% yield). ^1^H-NMR: (CDCl3) δ (ppm): 4.771–4.871 (4H, d, =CH2), 3.480 (4H, s, -CH2), 2.201 (6H, s, -CH3).

The synthetic route is shown as follows:

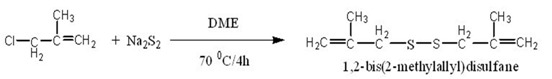



### 3.3. Cell culture

HepG2 cells, a human hepatoma cell line, was cultured at 37 ºC in DMEM with 10% heat-inactivated fetal bovine serum (FBS), benzylpenicillin (100 kU/L) and streptomycin (100 mg/L) in an incubator containing humidified air with 5% CO_2_.

### 3.4. Cell viability assay

Cells were seeded into 96-well plates at 1 × 10^4 ^cells per well 24 h before treatment. The cultures were then rinsed in phenol-free DMEM medium and incubated with respective test substances in phenol-free and serum free DMEM for 24 h. In the inhibition test, cells were treated with 1,2-bis(2-methylallyl)disulfane. At the end of this time interval, 20 μL (5 mg/mL) MTT (3-(4,5dimethylthiazol-2-yl)-2,5-diphenyltetrazolium bromide) was added to each well, and after incubation at 37 ºC for 4 h the MTT solution was removed and 200 μL of dimethylsulfoxide (DMSO) was added to dissolve the crystals. The absorbance of each well was measured at 570 nm.

### 3.5. Flow cytometry analysis

Cells were seeded into 100-mL cell culture bottles at 12 × 10^6^ cells 24 h before treatment. Then cells were treated according to the aforementioned method and incubated for 24 h. Afterwards, cells were collected, combined into single cell suspension and centrifuged at 800 ×g for 5 min. The supernatant was discarded and the cells were washed three times with cooled PBS and fixed for 24 h with cold alcohol at 4 ºC. 1 mL cell suspension (10^6^/mL) was washedken for3640364036403640364036403640364036403640364036403640364036403640364036403640364036403640364036403640364036403640364036403640364036403640364036403640364036403640364036403640364036403640364036403640364036403640364036403640364036403640364036403640364036403640364036403640364036403640364036403640364036403640364036403640364036403640364036403640364036403640364036403640364036403640364036403640364036403640364036403640364036403640364036403640364036403640364036403640364036403640 three times with the cool PBS, treated with RNase for 30 min at 37 ºC, stained it with PI for 30 min at 37 ºC in a dark environment, and taken for flow cytometry analysis.

### 3.6. Western-blotting

The cells were taken in the logarithmic growth phase, treated according to the aforementioned method and incubated for 24 h. After fragmentation on ice for 20 min, the lysates were centrifuged at 15,000 ×g for 10 min at 4 ºC, the protein was collected, quantitated with the BCA method, electrophoresed and isolated by the SDS-PAGE (10%) using the electrotransfer method, blocked and hybridized on the cellulose nitrate film. The the protein expression of cells was detected using the ECL Western blotting method. The densities of protein bands were calculated using the Quantity One software.

### 3.7. Statistics

Data are expressed as mean ±S.D of three independent experiments and were evaluated by one-way analysis of variance (ANOVA). Significant differences were established at P < 0.05.

## 4. Conclusions

1,2-bis(2-methylallyl)disulfane was prepared and characterized by GC-MS, ^1^H-NMR and elemental analysis. The cell viability assay, flow-cytometric analysis of apoptosis and protein expression showed that 1,2-bis(2-methylallyl)disulfane could significantly inhibit proliferation and induce apoptosis in a dose dependent manner in human HepG2 cells. 
